# 2-(4-Nitrophenyl)isothiazol-3(2H)-one: A Promising Selective Agent against Hepatocellular Carcinoma Cells

**DOI:** 10.3390/ph17060673

**Published:** 2024-05-24

**Authors:** Sofia Marka, Maria-Eleftheria Zografaki, Georgia Tsolomiti, Katerina I. Kalliampakou, Athanasios Tsolomitis, Christina Koumantou, Despina Smirlis, Niki Vassilaki, Spyros Kintzios

**Affiliations:** 1 Laboratory of Cell Technology, Department of Biotechnology, School of Applied Biology and Biotechnology, Agricultural University of Athens, 11855 Athens, Greece; georgia.tsolomiti@aua.gr (G.T.); christinakoumantou@gmail.com (C.K.); 2 Laboratory of Molecular Biology, Department of Biotechnology, School of Applied Biology and Biotechnology, Agricultural University of Athens, 11855 Athens, Greece; mzografaki@aua.gr (M.-E.Z.); kalliamp@aua.gr (K.I.K.); 3 Laboratory of Molecular Virology, Hellenic Pasteur Institute, 11521 Athens, Greece; nikiv@pasteur.gr; 4 School of Chemical Engineering, National Technical University, 15772 Athens, Greece; athtsolom@gmail.com; 5 Molecular Parasitology Laboratory, Microbiology Department, Hellenic Pasteur Institute, 11521 Athens, Greece; penny@pasteur.gr

**Keywords:** anticancer, cytotoxic, hepatocellular carcinoma, Huh7, isothiazolone, 2-(4-nitrophenyl)isothiazol-3(2H)-one, 5-benzoyl-2-(4-nitrophenyl)isothiazol-3(2H)-one

## Abstract

Liver cancer ranks among the most prevalent malignancies globally and stands as a leading cause of cancer-related mortality. Numerous isothiazolone derivatives and analogues have been synthesized and investigated for their potential as anticancer agents; however, limited data exist regarding their efficacy against liver cancer. In the present study, two nitrophenyl-isothiazolones, the 5-benzoyl-2-(4-nitrophenyl)isothiazol-3(2H)-one (IsoA) and the 2-(4-nitrophenyl)isothiazol-3(2H)-one (IsoB), were preliminarily investigated for their cytotoxicity against hepatoma human (Huh7) cells as a liver cancer model and Immortalized Human Hepatocytes (IHHs) as a model of non-cancerous hepatocytes. IsoB, derived from IsoA after removal of the benzoyl moiety, demonstrated the highest cytotoxic effect against Huh7 cells with CC_50_ values of 19.3 μΜ at 24 h, 16.4 μΜ at 48 h, and 16.2 μΜ at 72 h of incubation, respectively. IsoB also exhibited selective toxicity against the liver cancerous Huh7 cells compared to IHH cells, reinforcing its role as a potent and selective anticancer agent. Remarkably, the cytotoxicity of IsoB was higher when compared with the standard chemotherapeutical agent 5-fluorouracil (5-FU), which also failed to exhibit higher toxicity against the liver cancerous cell lines. Moreover, IsoB-treated Huh7 cells presented a noteworthy reduction in mitochondrial membrane potential (ΔΨm) after 48 and 72 h, while mitochondrial superoxide levels showed an increase after 24 h of incubation. The molecular mechanism of the IsoB cytotoxic effect was also investigated using RT-qPCR, revealing an apoptosis-mediated cell death along with tumor suppressor TP53 overexpression and key-oncogene MYCN downregulation.

## 1. Introduction

Isothiazole and analogues like isothiazolones have found applications in different fields since they have useful biological properties, such as antimicrobial, antibacterial, antifungal, antiviral, and anti-inflammatory activities [1,2]. Isothiazolones are organic compounds containing five-membered sulfur–nitrogen heterocyclic rings with various substitution patterns and functional groups attached to the core isothiazolone structure. They are known for their extensive useful biological activity as bactericides [3,4] in different industrial sectors and as active agents in many pharmaceuticals and agrochemicals [2]. Except from potent biocides, isothiazolones have been investigated as anticancer agents due to their demonstrated ability to disrupt key cellular processes involved in cancer progression [5,6,7,8,9]. Through targeted mechanisms, isothiazolones exhibit the potential to induce apoptosis and inhibit tumor growth, making them subjects of considerable interest in cancer research [10,11,12]. Various isothiazolone derivatives were tested on different cancer cell lines, including promyeoloblast cells (HL 60), Hodgkin’s Lymphoma cells (L428), human lung adenocarcinoma (A549), hepatocellular carcinoma (HepG-2), colorectal carcinoma (HCT-116), and breast cancer (MCF-7) cells, and their results revealed that these compounds exhibited significant cytotoxicity, antitumor efficacy, induction of necrosis, as well as antibacterial and anti-inflammatory properties [10,13,14,15].

Liver cancer stands among the leading causes of global cancer deaths, ranking as the sixth most frequently diagnosed cancer globally and the fourth primary contributor to cancer-related mortality [16]. The frequency of liver carcinomas is on the rise in developed countries, although the majority of liver cancer cases occur in developing countries [17,18]. In 2020, an estimated 905,700 people were diagnosed, and 830,200 people died from liver cancer globally, rendering the disease among the top three causes of cancer death in 46 countries and among the top five causes of cancer death in 90 countries [19]. Hepatocellular carcinoma (HCC) is the most common form of liver cancer, accounting for over 80% of all cases and occurring in >90% of cases in patients with liver damage. It has an estimated 18% 5-year survival rate [16]. The treatment option for HCC is surgical resection, which, however, is only limited to patients with good liver function at the early stage of the disease but has a high relapse rate (>60%) after surgery [17]. The number of new cases of liver cancer per year is predicted to increase by 55.0% by 2040, leading to 1.3 million deaths [19]. 

Liver cancer is linked to dysregulated signaling pathways and various genetic changes, encompassing the inactivation of tumor suppressors, activation of oncogenes, and dysregulation of genes related to apoptosis, cell proliferation, survival, inflammation, angiogenesis, telomerase reactivation, and cell metabolism [20,21]. The existing molecular targeted therapeutic strategies for treating hepatocellular carcinoma involve the utilization of various agents, such as tyrosine kinase inhibitors (TKIs) like sorafenib and lenvatinib [22,23], antimetabolites such as 5-fluorouracil (5-FU) [24,25], and immune checkpoint inhibitors (ICIs), including monoclonal antibodies like Atezolizumab and Bevacizumab [26]. Despite ongoing progress, the current efficacy of HCC treatment is insufficient, prompting the need for an extensive exploration of novel agents or strategies. Although isothiazoles have shown promise as potential anticancer drugs in various cancer types, there is limited literature available regarding their efficacy, specifically in liver cancer.

In the present study, the in vitro cytotoxic activity of two previously described and synthesized isothiazolones, 5-benzoyl-2-(4-nitrophenyl)isothiazol-3(2H)-one (IsoA) and 2-(4-nitrophenyl)isothiazol-3(2H)-one (IsoB) [27], was investigated. IsoA is a benzisothiazole compound and was chosen for the potent and extensively employed biocidal and anticancer activity of that type of isothiazolone [28,29,30], while IsoB derived from IsoA has a more simplified structure lacking the benzoyl moiety. The effects of these isothiazolones and of the standard chemotherapeutical agent 5-FU were tested concerning their cytotoxicity on epithelial-like, human hepatoma liver cancer cells (Huh7) and on IHH cells used as a model of non-cancerous hepatocytes. IsoB was found to exhibit considerable in vitro cytotoxicity compared to 5-FU against Huh7 cells, whereas the opposite was observed in the case of IHH cells. The underlined mechanism of the IsoB cytotoxic effects on Huh7 cells was investigated by analyzing effects on gene expression, as well as on the redox status of the cell and the mitochondrial membrane potential.

## 2. Results

### 2.1. Dose- and Time-Dependent Cytotoxic Effects of IsoA, IsoB, and 5-FU on Huh7 Cells

**Figure 1:**
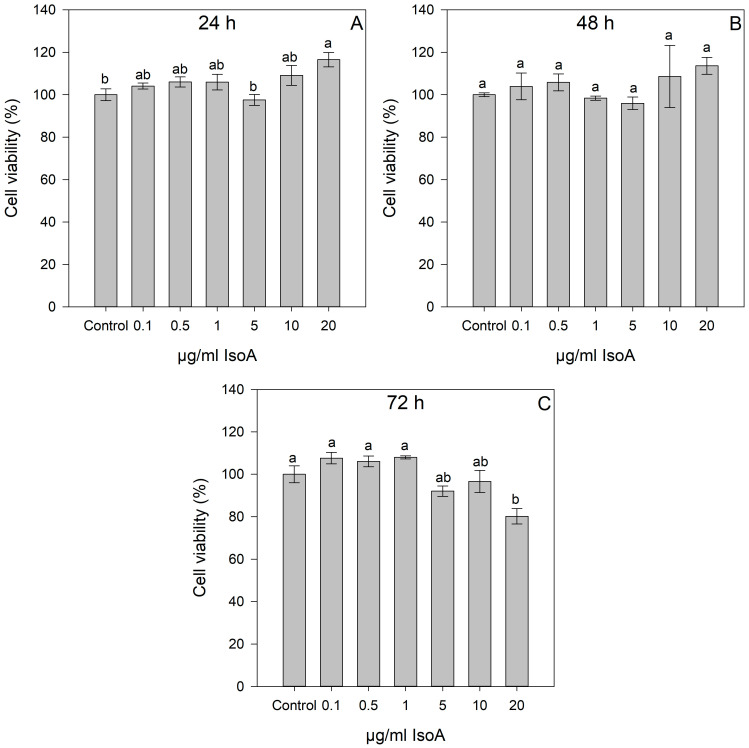
Dose- and time-dependent effects of IsoA on Huh7 cell viability after 24 h (**A**), 48 h (**B**), and 72 h (**C**) of treatment. Cell viability, assessed by the MTT assay, is expressed relative to control (non-treated) cells. The viability of control cells was set to 100%. Bars represent mean values, and error bars represent standard errors (n = 5). Different letters above bars represent significant changes in cell viability among different concentrations of IsoA used, as determined by Tukey’s test (*p* < 0.05).

**Figure 2:**
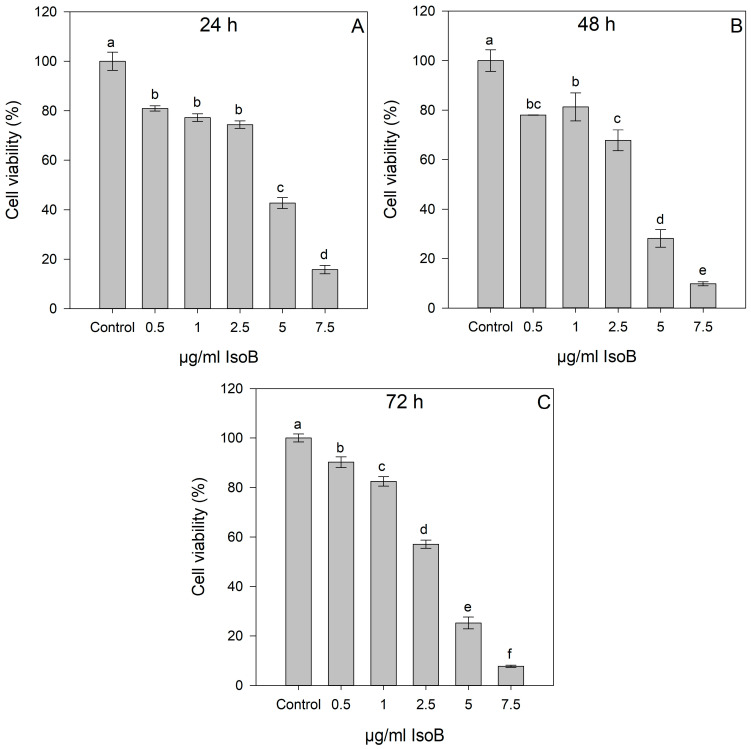
Dose-dependent effect of isothiazolone IsoB on Huh7 cell viability after 24 h (**A**), 48 h (**B**), and 72 h (**C**) of treatment. Cell viability, assessed by the MTT assay, is expressed relative to control cells. The viability of control cells was set to 100%. Bars represent mean values, and error bars represent standard errors (n = 5). Different letters above bars represent significant changes in cell viability among different concentrations of IsoB used, as determined by Tukey’s test (*p* < 0.05).

**Figure 3:**
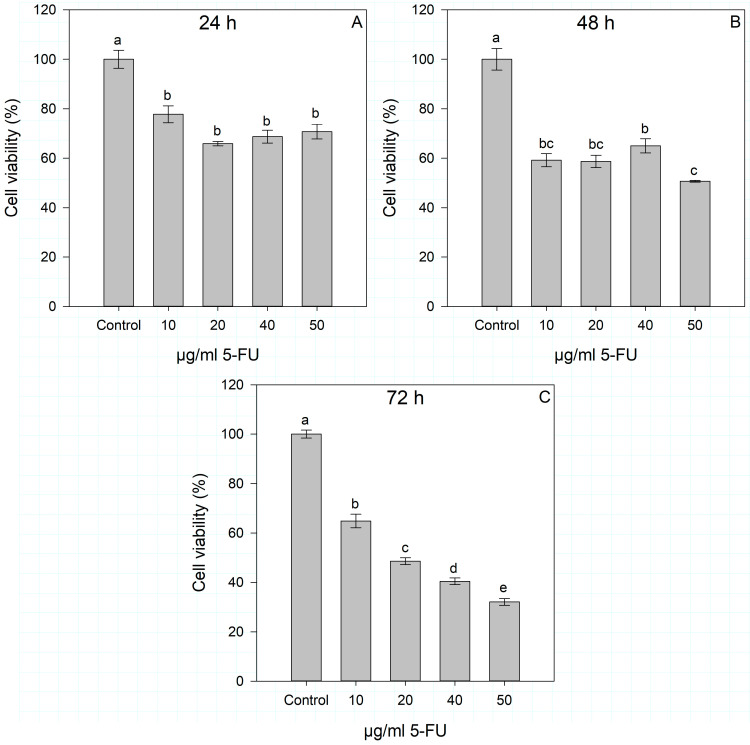
Dose-dependent effects of 5-FU on Huh7 cell viability after 24 h (**A**), 48 h (**B**), and 72 h (**C**) of treatment. Cell viability is expressed relative to control cells. The viability of control cells was set to 100%. Bars represent mean values, and error bars represent standard errors (n = 5). Different letters above bars represent significant changes in cell viability among different concentrations of 5-FU used, as determined by Tukey’s test (*p* < 0.05).

The previously synthesized nitrophenyl-isothiazolones, IsoA and IsoB, were tested on the Huh7 hepatocellular carcinoma cell line to assess their cytotoxic effects. The 3-(4,5-dimethyldiazol-2-yl)-2,5-diphenyl-2H-tetrazolium bromide (MTT) assay was used to detect the cytotoxic effects of different doses of IsoA, IsoB, and 5-FU on Huh7 cells, and the results are depicted in Figure 1, Figure 2, and Figure 3, respectively. As shown, all tested concentrations (0.1–20 μg/mL) of IsoA were generally well tolerated by the Huh7 cells as slightly lower viability was observed only at the highest dose (20 μg/mL) after 72 h of treatment (Figure 1). 

IsoB treatment induced high rates of cell death in Huh7 cells at concentrations of 2.5 to 7.5 μg/mL compared to the control cells. Cytotoxic effects were IsoB dose-dependent and observed even after 24 h of treatment (Figure 2). 

In addition, the dose- and time-dependent effects of the standard chemotherapeutical agent 5-FU were evaluated on Huh7 cells after 24, 48, and 72 h of incubation compared to control cells. Different concentrations of 5-FU (10–50 μg/mL) were tested according to the literature [31,32,33], and the results of cell viability are shown in Figure 3. Significant differences between the control and 5-FU-treated cells were observed at all time points (24, 48, and 72 h) and at all concentrations used, but 5-FU manifested strong effects only after 72 h of treatment and at concentrations of 20 to 50 μg/mL. A dose-dependent effect of 5-FU was observed only after 72 h of incubation, and the 50% cytotoxic concentration (CC_50_) was calculated by linear regression. The calculated equation was y = −0.737x + 68.612 (R^2^ = 0.9274), which resulted in CC_50_ of 25.25 μg/mL 5-FU for Huh7 cells.

**Figure 4:**
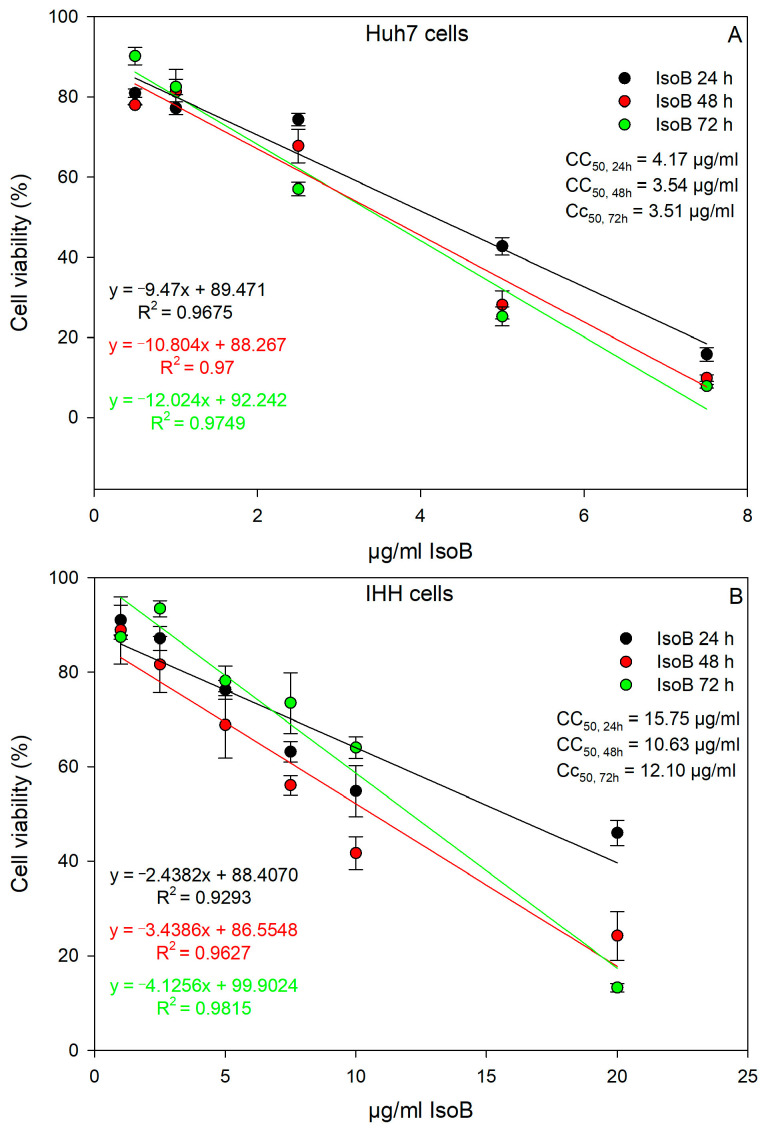
Dose-response curves of IsoB on Huh7 (**A**) and IHH (**B**) cell viability after incubation for 24 h (black), 48 h (red), and 72 h (green). Cell viability is expressed as a percentage relative to control cells, the viability of which was set to 100%. Values are expressed as means ± SE (n = 5).

Thus, IsoB appears to be superior in its cytotoxic effects on Huh7 cancer cells compared to 5-FU. The relationship between IsoB concentration and Huh7 cell viability was analyzed by linear regression to determine its CC_50_ for the three different incubation times of treatment (24 h, 48 h, 72 h). At 24 h of treatment, the CC_50_ value was 4.17 μg/mL (19.3 μΜ); at 48 h, the CC_50_ was 3.54 μg/mL (16.4 μM); and at 72 h, the CC_50_ was 3.51 μg/mL (16.3 μM) (Figure 4A). Moreover, CC_30_ and CC_70_ were also calculated from the dose-response curve linear equations, resulting in 2.06 μg/mL and 6.28 μg/mL for 24 h, 1.69 μg/mL and 5.39 μg/mL for 48 h, and 1.85 μg/mL and 5.18 μg/mL for 72 h, respectively, for IsoB treatment of Huh7 cells. IsoB was also tested on non-cancerous human hepatocytes (IHHs), and the CC_50_ was calculated for three different incubation times of treatment (24 h, 48 h, 72 h) to investigate its potential selective anticancer activity (Figure 4B). The CC_50_ values of IsoB on IHH cells were 15.75 μg/mL for 24 h, 10.63 μg/mL for 48 h, and 12.10 μg/mL for 72 h. These values were found to be three- to four-fold higher than those observed in the case of Huh7 cells, highlighting IsoB’s selective cytotoxicity against liver cancerous cells.

**Figure 5:**
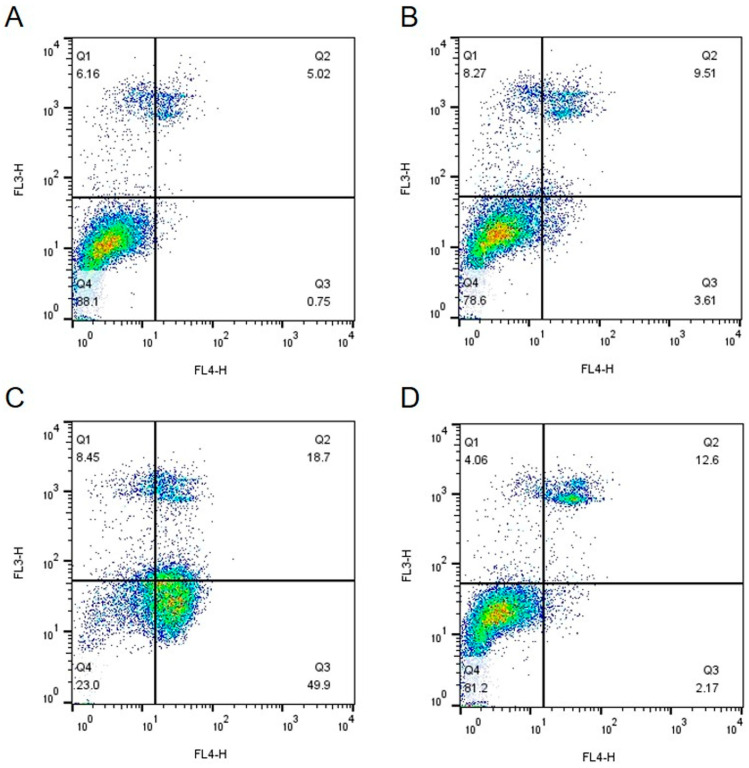
FACS analysis of Huh7 cells to determine the effects of IsoB and 5-FU on cell viability at 48 h of incubation. Dead cells are positive for propidium iodide staining detected by using FL3-H filter (Q1, Q2); early apoptotic cells are positive only for allophycocyanin-conjugated annexin V staining detected by using FL4-H (Q3); healthy cells are negative for both staining methods (Q4). Huh7 control cells (**A**), cells treated for 48 h with the respective IsoB CC_30_ (**B**), cells treated for 48 h with the respective IsoB CC_50_ (**C**), and cells treated with 10 μg/mL 5-FU (**D**). The dot plots shown are non-gated, and the same central vertex has been used to determine the quadrants in all plots. For all experiments, 10,000 events were collected and analyzed. A representative experiment from three independent repetitions is shown.

**Figure 6:**
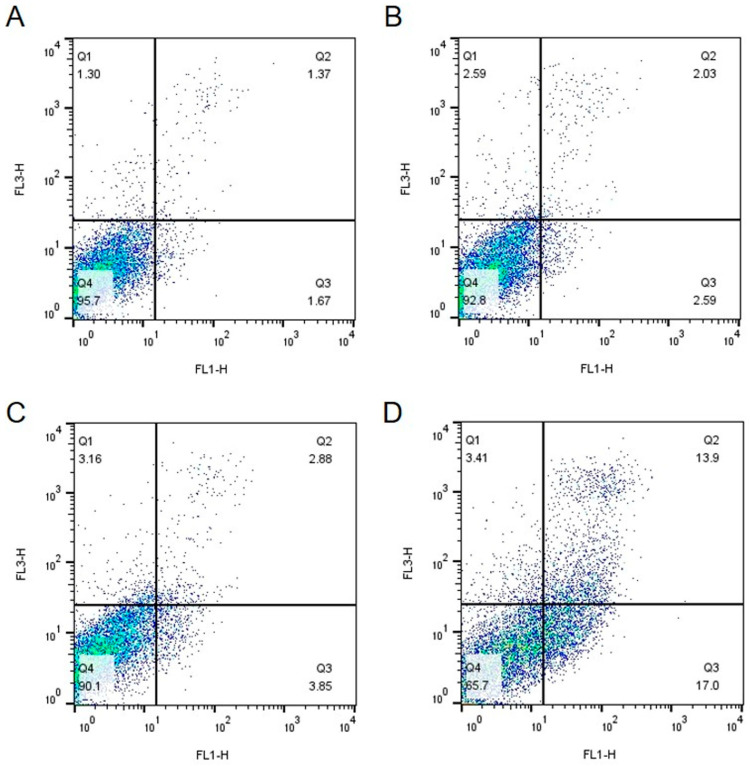
FACS analysis of IHH cells to determine the effects of IsoB and 5-FU on cell viability at 48 h of incubation. Dead cells are positive for propidium iodide staining detected by using FL3-H filter (Q1, Q2); early apoptotic cells are positive only for 488 annexin V staining detected by using FL1-H (Q3); healthy cells are negative for both staining methods (Q4). IHH control cells (**A**), cells treated for 48 h with the respective IsoB CC_30_ (**B**), cells treated for 48 h with the respective IsoB CC_50_ (**C**), and cells treated with 10 μg/mL 5-FU (**D**). The dot plots shown are non-gated, and the same central vertex has been used to determine the quadrants in all plots. For all experiments, 10,000 events were collected and analyzed. A representative experiment from three independent repetitions is shown.

The effects of IsoB and 5-FU on Huh7 and IHH cells were also analyzed using Fluorescence-Activated Cell Sorting (FACS) analysis. For this, cells were incubated for 48 h with the respective IsoB CC_30_, CC_50_ concentrations, 10 μg/mL 5-FU, or were not treated (control cells). At 48 h of incubation, the cells were collected and double stained with propidium iodide and Annexin V and then subjected to FACS analysis. The results acquired from Huh7 cells are shown in Figure 5, and those obtained by IHH cells are depicted in Figure 6.

As shown, the use of IsoB resulted in elevated rates of cell death in Huh7 cells, as indicated by the presence of a high percentage of annexin-positive cells and dual annexin and propidium iodide positive staining cells, especially in the case of IsoB CC_50_ concentration (Figure 5C). Interestingly, the same IsoB concentration, when applied for 48 h to IHH cells, had only slight effects on cell viability (Figure 6C). On the opposite, 5-FU appeared to induce higher rates of apoptosis in non-cancerous IHH cells compared to cancerous Huh7 cells (Figure 5D and Figure 6D). The above results suggested that IsoB may have a selectivity against liver cancer cells as compared to normal hepatocytes. 

### 2.2. IsoB-Induced Effects on Membrane Potential and Mitochondrial Superoxide Production

**Figure 7:**
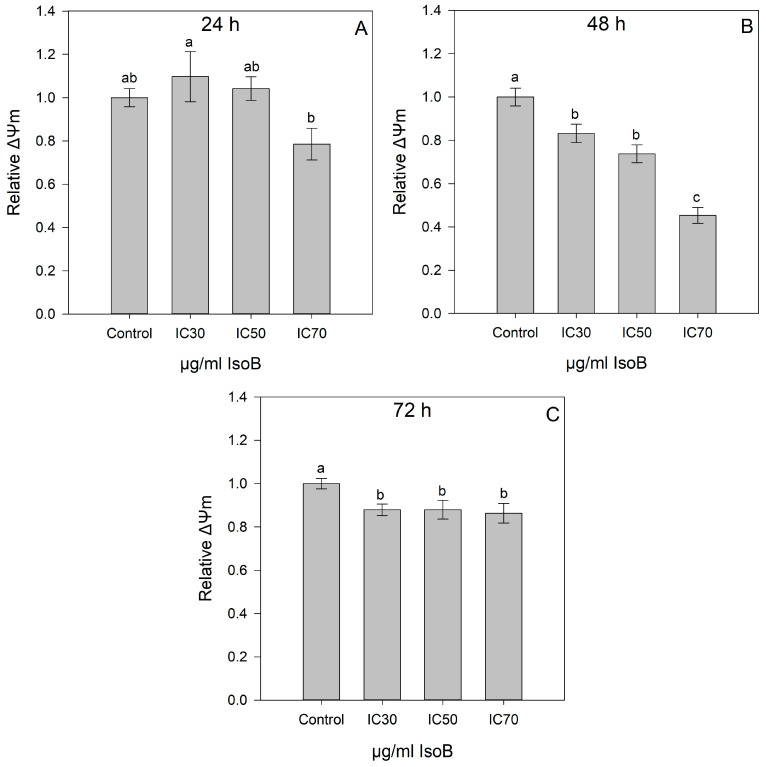
Assessment of mitochondrial membrane potential (ΔΨm) of Huh7 cells after 24 h (**A**), 48 h (**B**), and 72 h (**C**) of incubation with the respective CC_30_, CC_50_ and CC_70_ of IsoB. Staining of the cells was performed with MitoTracker Red and Green fluorescent dyes. Control cells were not treated with IsoB (control). Relative ΔΨm was calculated by the fluorescence ratio of MitoTracker Red, which stains mitochondria based on their membrane potential, to MitoTracker Green, which stains mitochondria independent of their membrane potential, and normalized to the respective ratio of control cells. Bars represent mean values, and error bars represent standard errors (n = 5). The mitochondrial membrane potential (ΔΨm) of control cells was set at 1. Different letters above bars represent significant changes in mitochondrial membrane potential (ΔΨm) among the different concentrations of IsoB used, as determined by Tukey’s test (*p* < 0.05).

Cell-permeable mitochondrial staining dyes were used to assess the mitochondrial membrane potential of Huh7 cells treated with different concentrations of IsoB (CC_30_, CC_50_, and CC_70_). The mitochondrial membrane potential (ΔΨm) was assessed in Huh7 cells treated with IsoB for 24 h, 48 h, and 72 h using the respective CC_30_, CC_50_, and CC_70_ concentrations (Figure 7). As shown, upon the 24-h treatment, no statistically significant differences were observed in the mitochondrial membrane potential among the different concentrations of IsoB (CC_30_, CC_50_, CC_70_) used and the control cells. However, for the 48-h treatment, the results revealed a gradual decrease in mitochondrial membrane potential for the respective CC_30_, CC_50_, and CC_70_ values. Moreover, the 72-h treatment of Huh7 cells with the respective CC_30_, CC_50_, and CC_70_ resulted in a slight decrease in mitochondrial membrane potential compared to control cells.

**Figure 8:**
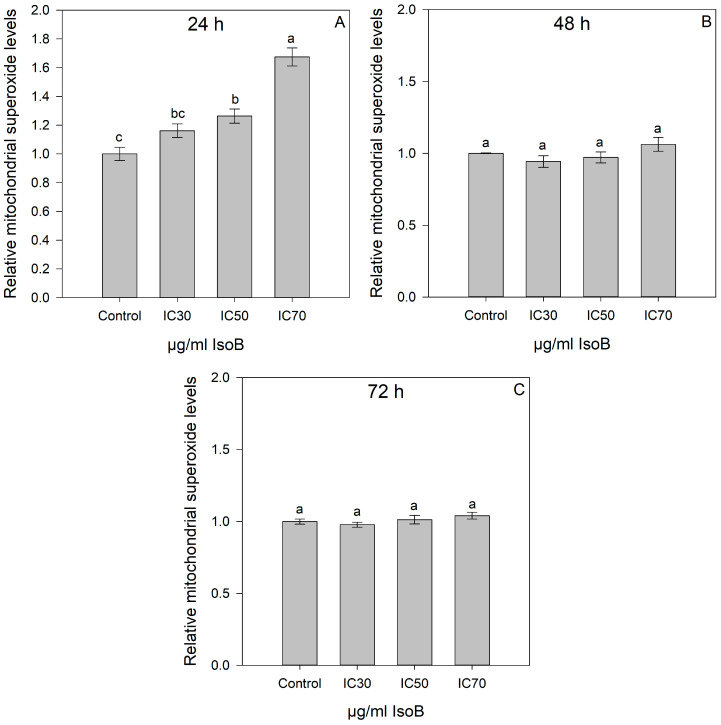
Measurements of mitochondrial superoxide levels in Huh7 cells after incubation with the respective CC_30_, CC_50_, and CC_70_ of IsoB for 24 h (**A**), 48 h (**B**), and 72 h (**C**), respectively. Staining of the cells was performed with MitoSOX Red and MitoTracker Green fluorescent dyes. Control cells were not treated with IsoB (control). Relative mitochondrial superoxide levels were determined based on the fluorescence ratio of MitoSOX Red to MitoTracker Green values and were normalized to that of the control cells. Bars represent mean values, and error bars represent standard errors (n = 5). The mitochondrial superoxide level of control cells was set at 1. Different letters above bars represent significant changes in mitochondrial superoxide levels among different concentrations of IsoB used, as determined by Tukey’s test (*p* < 0.05).

In addition, the effects of the respective CC_30_, CC_50_, and CC_70_ of IsoB for 24-h, 48-h, and 72-h treatment of Huh7 cells on mitochondrial superoxide production were determined using the live-cell permeant superoxide indicator, MitoSOX (Figure 8). Notably, for the 24-h treatment, a significant increase in Huh7 superoxide levels was observed at CC_50_ and CC_70_ concentrations of IsoB as compared to the control cells. However, no significant differences were observed in superoxide levels of Huh7 cells treated with the respective CC_30_, CC_50_, and CC_70_ concentrations of IsoB for 48 h and 72 h compared to the control cells. 

### 2.3. Gene Expression of IsoB-Treated Huh7 Cells

**Figure 9:**
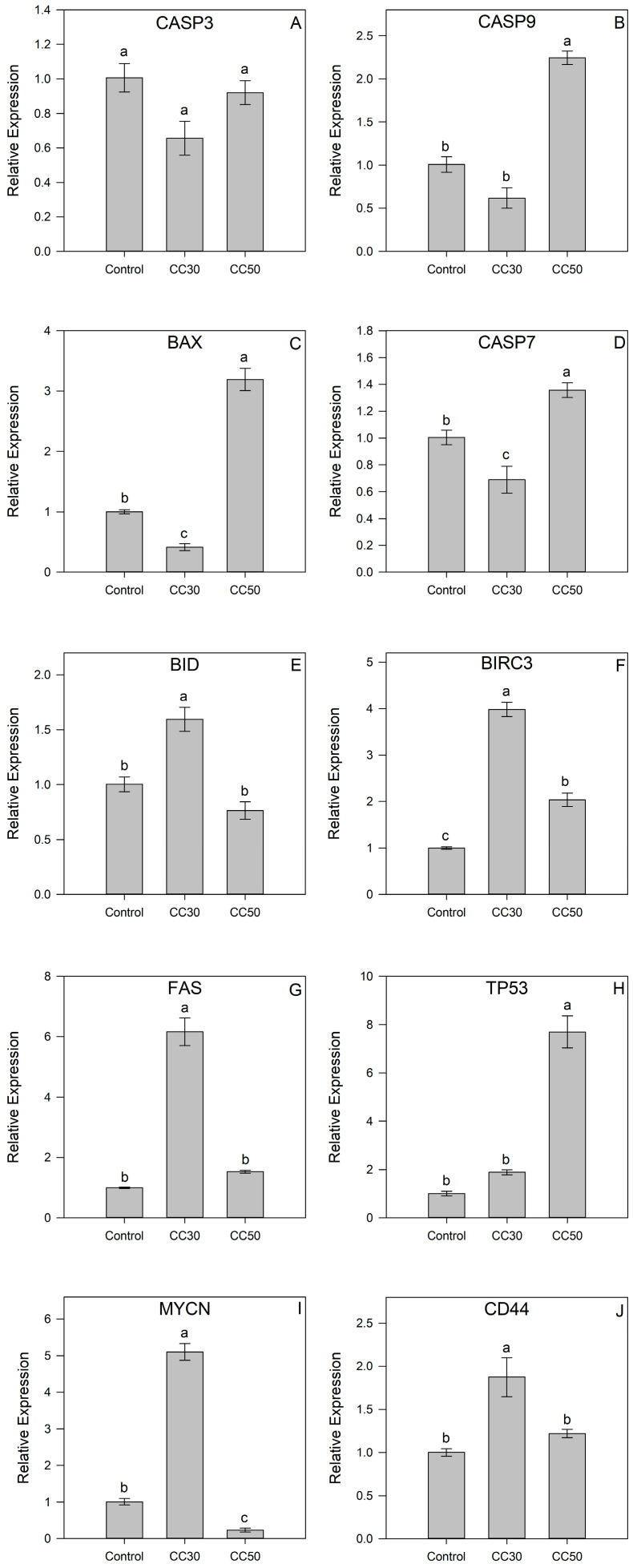
Relative gene expressions of Huh7 cells after treatment for 48 h with the respective CC_30_ and CC_50_ concentrations of IsoB. Cells were incubated with two concentrations of IsoB corresponding to CC_30_ and CC_50_, and relative expressions were evaluated for the genes: *CASP3* (**A**), *CASP9* (**B**), *BAX* (**C**), *CASP7* (**D**), *BID* (**E**), *BIRC3* (**F**), *FAS* (**G**), *TP53* (**H**), *MYCN* (**I**), and *CD44* (**J**). Values are mean ± SE (n = 3). Different letters above bars represent significant changes in gene expression among different concentrations of IsoB used, as determined by Tukey’s test (*p* < 0.05).

To gain a deeper insight into the molecular mechanism of the effect of IsoB on Huh7 cells, the transcript accumulation of several target genes involved in cellular apoptosis and cancer-related signaling was studied after extraction of total RNA, reverse transcription, and quantitative PCR (RT-qPCR). In particular, Huh7 cells were treated with two concentrations of IsoB corresponding to the respective CC_30_ and CC_50_ of 48-h treatment, and the relative gene expression is depicted in Figure 9. The apoptotic gene BCL2-associated X protein (BAX) was significantly upregulated up to three-fold in Huh7 cells treated with CC_50_ of IsoB compared to the control cells, whereas it was two-fold downregulated in cells treated with CC_30_. On the other hand, the incubation of Huh7 cells with the IsoB CC_30_ concentration resulted in a significant upregulation of BH3 interacting domain death agonist (BID) in comparison to control cells, while there was no significant difference in IsoB CC_50_-treated cells. A similar transcript accumulation pattern was observed in the case of Fas cell surface death receptor (FAS), where Fas transcripts were found to be six-fold higher in CC_30_-treated cells, and no difference was revealed in CC_50_-treated cells. In addition, transcripts of baculoviral IAP repeat containing (BIRC3) were found to be four-fold higher in IsoB CC_30_-treated Huh7 cells and two-fold upregulated in IsoB CC_50_-treated cells. Furthermore, the transcript accumulations of three genes encoding cysteine proteases with pivotal roles in apoptosis, caspase 3 (CASP3), caspase 7 (CASP7), and caspase 9 (CASP9) were studied. The treatment with the two concentrations (CC_30_ and CC_50_) of IsoB did not alter the expression of CASP3 compared to control cells. However, the transcripts of both CASP7 and CASP9 were found to be significantly higher in IsoB CC_50_-treated Huh7 cells, whereas CASP7 expression was downregulated in CC_30_-treated cells. Interestingly, the MYCN proto-oncogene, bHLH transcription factor (MYCN), was five-fold upregulated in CC_30_-treated cells, while it was downregulated in CC_50_-treated cells. In contrast, tumor protein p53 (TP53) transcripts were found to be more than seven-fold higher only in the Huh7 cells incubated with the high concentration of IsoB (CC_50_). Finally, CD44 transcript accumulation was significantly higher in IsoB CC_30_-treated cells, whereas there was no difference observed in CC_50_-treated cells.

## 3. Discussion

Even though isothiazolones are widely used as preservative additives in cosmetics and industrial chemicals, their biocidal properties have attracted the attention of cancer researchers, and their in vitro tumor cytotoxic effects have been documented in several studies. 

However, the literature on the utilization of isothiazolones as anticancer agents in liver cancer is limited. In a study by Arning J. et al. [34], human hepatocarcinoma cells were used as a model system to assess the structure–activity relationships of four distinct isothiazol-3-one derivatives on cellular glutathione metabolism, which is highly elevated in many tumor cases. The study revealed that the derivatives notably reduced total cellular glutathione levels in a concentration- and time-dependent manner. Moreover, all examined isothiazol-3-ones led to elevated cellular levels of GSSG in a concentration- and time-dependent manner. Additionally, concentration–response curves indicated high toxicity for the studied derivatives of isothiazol-3-one, with a half-lethal concentration of 130 μM observed in the HepG2 liver cancer cell line [35]. Dekker et al. synthesized different isothiazolones and investigated their role in histone modifications and acetylation, which are implicated in several cancer types, including HCC. The isothiazolone derivative showed a significant effect on histone acetyltransferase (HAT) activity, with a half-maximal inhibitory concentration of less than 10 μM in HepG2 cells, although cell growth was not inhibited [36]. Gorsuch et al. [37] described two more isothiazoles as HAT inhibitors at concentrations ranging from 1.5 to 28 μM, depending on different substituents. Furthermore, Hayakawa et al. [7] emphasized the potential of telomerase-targeted antitumor drugs and introduced an isothiazolone derivative as a selective telomerase inhibitor in a rat model of liver cancer.

Isothiazolones, therefore, represent a group of promising candidates as cancer chemotherapeutical agents. In this context, the present study provides new data that concern the bioactivity of two synthesized isothiazolones, IsoA and IsoB, produced by the same chemical synthesis pathway, with IsoA being the precursor of IsoB. In our study, we investigated the effect of IsoA and IsoB on the Huh7 cell line used as a model of human hepatocellular carcinoma, and our results revealed intriguing findings with significant implications for potential therapeutic applications. It was observed that the synthesized IsoA compound containing the benzοyl moiety demonstrated a cytotoxic effect on Huh7 cells only when used at high concentrations (20 μg/mL) and only after prolonged exposure (72 h) of cells to this concentration. In contrast, IsoB, derived from IsoA by removing the benzoyl moiety, exhibited a pronounced dose-dependent cytotoxic effect on Huh7 cells across all time points even at low concentrations (0.5–7.5 μg/mL). The effectiveness of IsoB was further underscored when compared to the standard chemotherapeutic agent 5-FU, which displayed significantly lower cytotoxicity when used at similar concentrations (10 μg/mL) to that of IsoB. This suggests a potential advantage of IsoB in achieving cytotoxic effects at substantially lower concentrations and within a shorter timeframe. In addition, the CC_50_ value for IsoB falls well within the lower part of the range of respective concentrations of other isothiazolones determined for their in vitro cancer cytotoxicity [15,38,39]. In particular, the observed effect on cell viability of IsoB with 3.54 μg/mL CC_50_ (16.3 μM) is comparable to those reported by Lu et al. [40] and Zaharia et al. [14] against various cancer lines. Indeed, as far as Huh7 is concerned, the previously determined CC_50_ values range between 0.41 and 50 μΜ, depending on the specific substitution in the central thiazole ring. Additionally, when IsoB was tested on Immortalized Human Hepatocytes (IHH), the compound exhibited significantly lower cytotoxicity with CC_50_ values that were 3–4 times higher for all tested incubation times. These findings underscore the substantial potential of IsoB in HCC treatment. The above results of cell viability assays were in accordance with that of FACS analysis, which additionally revealed the property of IsoB to induce high rates of apoptosis to the cancerous Huh7 cell line as opposed to non-cancerous hepatocytes. In contrast, 5-FU induced higher rates of apoptosis in IHH cells compared to cancerous Huh7 cells. Thus, IsoB appears to be superior to 5-FU in selectively killing liver cancer cells. 

Of particular interest is the observation that the loss of the benzοyl moiety at the 5-position of IsoB resulted in a considerable reduction of the in vitro cytotoxic effect of its derivative IsoA, although benzoyl derivatives such as benzoyl peroxide are known for their antibacterial and cross-species cytotoxic properties [41]. There is scarce information on similar effects in the literature. For example, Perinelli et al. [42] reported a considerably higher antimicrobial activity and toxicity of the synthesized quats surfactants upon removal of their benzoyl group. 

Mitochondria play a crucial role in hepatocyte physiology, actively participating in cell energy production, metabolism, regulation, and survival. Mitochondrial transformations are integral to cancer development and progression [43]. Cancer cells reprogram mitochondrial energy metabolism and signaling to meet their heightened biosynthetic demands, thereby promoting cancer cell growth and survival, even in the face of environmental limitations, including therapeutic strategies [44]. Thus, many studies highlight mitochondria as a susceptible target for cancer therapy [45]. In this study, IsoB exhibited a potent effect against mitochondrial integrity, notably causing a significant decrease in membrane potential (ΔΨm) after 48 h of incubation and an increase in superoxide levels after 24 h of incubation in Huh7 cells. In other studies, isothiazolone derivatives have similarly demonstrated effects of inducing mitochondrial dysfunction in the brain [46,47], bronchial cells [48], and primary vascular muscle cells [49], underscoring their potential role in cancer therapy.

Chemoresistance is a common and quite serious problem in the successful treatment of HCC [50]. Resistance to 5-FU has been associated with various factors, including the overexpression of the TRL4 Toll-like receptors (TLRs) [51] and the Kelch-like ECH-associated protein 1 (Keap1)–nuclear factor erythroid 2-related factor 2 (Nrf2) signaling pathway [52]. Hence, there is an apparent necessity to develop new drugs that could overcome HCC chemoresistance through a novel mode of action. While 5-FU, acting as an antimetabolite, can inhibit rRNA and tRNA processing and modification [53], little is yet known about the molecular cytotoxic mechanism of the isothiazolones. 

Based on the results of FACS and gene expression analyses in the present study, depending on the dose and time of treatment, cell apoptosis is induced, and many apoptosis-related genes are triggered in Huh7 cells. In particular, the incubation with IsoB CC_50_ concentration triggered apoptosis in Huh7, as shown by FACS analysis in accordance with the induction of several apoptosis-related genes, such as the pro-apoptotic *BAX*, *BID*, the receptor *FAS*, *CD44*, as well as *CASP7* and *CASP9*, which are associated with the execution of apoptosis [54]. Furthermore, liver cancer therapy often centers on targeting *TP53*, as this gene encodes a cell cycle regulator (p53) that is widely dysfunctional in cancer cases. The tumor suppressor protein p53 acts by regulating genes associated with key cellular processes, including cell cycle arrest, DNA repair, apoptosis, and autophagy. Consequently, the pharmacological modulation of p53 activity presents a promising strategy, supported by numerous ongoing clinical trials aimed at targeting negative regulators of *TP53* or reactivating mutant p53 [55,56,57]. Interestingly, while mutation-related inactivation of p53 is common in HCC, the Huh7 cell line exhibits a mutated codon leading to p53 overexpression [58]. Treatment with IsoB significantly increases the accumulation of *TP53* transcripts, underscoring the potential of IsoB in HCC therapy [59,60]. Moreover, targeting oncogenes or oncoproteins presents further therapeutic opportunities for the treatment of cancer. Oncogenes play a pivotal role in regulating fundamental cellular processes, such as growth, proliferation, and division. Current studies highlight *MYCN*, one of the most extensively studied cancer-related genes with a crucial role in the initiation, sustenance, and advancement of liver cancer, as a potential novel therapeutic and diagnostic target for hepatocellular carcinoma treatment [61,62,63]. The observed significant decrease in the oncogene *MYCN* expression in IsoB CC_50_-treated Huh7 highlights the potential use of IsoB in liver cancer treatment. The aforementioned effects of IsoB in the expression of cancer-relative genes emphasize its effect on crucial molecular pathways and biomarker genes in cancer pathology.

## 4. Materials and Methods

### 4.1. Chemicals and Reagents

Dullbecco’s modified Eagle’s medium (DMEM) was purchased from Gibco (Invitrogen, Carlsbad, CA, USA) and fetal bovine serum (FBS) from PAN Biotech (Aidenbach, Germany). The 3-[4,5-dimethylthiazol-2-yl]-2,5-diphenyltetrazolium bromide (MTT) and the 5-fluorouracil (5-FU) reagents were purchased from Sigma (St. Louis, MO, USA). MitoTracker™ Red, MitoTracker™ Green, dimethyl sulfoxide (DMSO), and phosphate-buffered saline (PBS) were purchased from Thermo Fisher Scientific (Waltham, MA, USA). MitoSOX Red, SYBR Select Master Mix, and Super-Script II were purchased from Invitrogen (Carlsbad, CA, USA).

#### 4.1.1. Isothiazolones

**Scheme 1:**
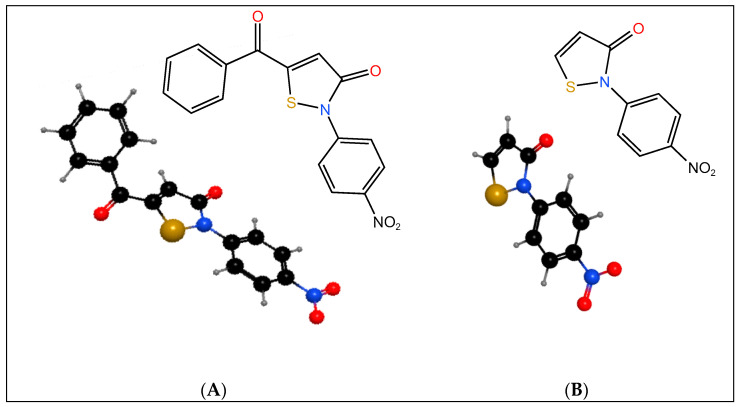
2D and 3D chemical structures of IsoA (**A**) and IsoB (**B**).

The two isothiazolones, 5-benzoyl-2-(4-nitrophenyl)isothiazol-3(2H)-one, IsoA (Scheme 1A), and 2-(4-nitrophenyl)isothiazol-3(2H)-one, IsoB (Scheme 1B), are shown. Both isothiazolones were synthesized from the same reaction sequence, and IsoB results from IsoA by removing the benzoyl moiety [27].

#### 4.1.2. Cell Culture

Human hepatocellular carcinoma cell line (Huh7) was purchased from Sigma–Aldrich, and Immortalized Human Hepatocytes (IHHs) were obtained from ATCC. Both cells were maintained in DMEM supplemented with 10% FBS and 1% penicillin/streptomycin in a humidified incubator with 5% CO_2_ at 37 °C. The cells were subcultured three times weekly. The 0.25% trypsin/0.2% EDTA solution was routinely used to detach the cells prior to seeding.

#### 4.1.3. Cell Viability Assessment

In vitro cytotoxicity of isothiazolone compounds (IsoA, IsoB) and 5-FU in Huh7 and IHH cells was evaluated by the MTT assay. In brief, cells (10^4^ cells/well) were seeded in 96-well culture plates and incubated overnight. After the removal of the medium, cells were treated with various concentrations of IsoA, IsoB, and 5-FU compounds dissolved in DMEM medium supplemented with 1% FBS and 1% penicillin/streptomycin incubated for 24 h, 48 h, and 72 h at 37 °C and 5% CO_2_. Cells were washed with PBS, and MTT solution (5 mg/mL) was added for 3 h incubation. The resulting purple formazan crystals of MTT were dissolved in pure DMSO, and absorbance was measured at 560 nm using a microplate reader (Infinite M200 PRO, Tecan, Männedorf, Switzerland). Relative viability was calculated after subtracting the background signal and normalizing it to the control cell. The concentrations of IsoB corresponding to CC_30_, CC_50_, and CC_70_ were calculated from the dose–response curves.

#### 4.1.4. Fluorescence-Activated Cell Sorting (FACS) Analysis

In the case of Huh7 cells, the APC Annexin V Kit (Biotium, Fremont, CA, USA, San Francisco Bay Area) was used to stain the apoptotic cells far red by the allophycocyanin-conjugated Annexin V. Propidium Iodide (PI) was used to stain necrotic cells red. In the case of IHH cells, the CF^®^488A Annexin V and PI Apoptosis Kit (Biotium, San Francisco Bay Area) were used to stain the apoptotic cells green and the necrotic cells red. 

Huh7 and IHH cells were incubated for 48 h with the respective IsoB CC_30_ or CC_50_ concentrations, with 10 μg/mL 5-FU, or were not treated (control cells). At 48 h of incubation, the cells were detached by trypsinization. For each treatment, the cell supernatant (growth medium), cell washes (PBS), cell suspension resulting from trypsin treatment, and well washes (PBS) were collected into a 15 mL tube. Then, each tube was centrifuged to precipitate cells, which after washing (PBS) and new centrifugation, were resuspended in 100 μL 1X Binding Buffer of the above Annexin V Kits. Then, the cells were stained using the kits mentioned above according to the manufacturer’s instructions, and after incubation for 15 min on ice, the cells were analyzed on FACS Calibur (Becton Dickinson, Hong Kong, China) using the appropriate channels. For each experiment, 10,000 events were collected and analyzed using Flow Jo (Miltenyi, Westphalia, Germany) and CELLQuest™ (Becton-Dickinson) software version 3.1. Three independent experiments were performed, and the results presented are from one representative experiment.

#### 4.1.5. Mitochondrial Membrane Potential (ΔΨm)

Huh7 cells (at a density of 10^4^ cells per well) were cultured in 96-well plates and treated for 24, 48, and 72 h with the corresponding concentration of IsoB (CC_30_, CC_50_, and CC_70_) in DMEM medium supplemented with 1% FBS and 1% antibiotics. Then, the cells were washed with PBS and stained with 100 nΜ MitoTracker™ Red (Thermo Fisher Scientific, Waltham, MA, USA) and 100 nΜ MitoTracker™ Green (Thermo Fisher Scientific, USA) for 30 min at 37 °C. Subsequently, after washing cells with PBS, fresh medium was added to each well, and fluorescence intensity was recorded at 490/516 nm (ex/em) and 581/644 nm (ex/em) using a fluorescence microplate reader (Infinite M200 PRO, Tecan). Mitochondrial membrane potential (ΔΨm) was evaluated by calculating the red/green fluorescence ratio [64].

#### 4.1.6. Mitochondrial Superoxide Levels

Detection of mitochondrial superoxide levels in Huh7 cells treated with IsoB was measured using MitoSOX Red (Invitrogen). Briefly, Huh7 cells (10^4^ cells/well) were treated for 24, 48, and 72 h with the corresponding concentration of IsoB (CC_30_, CC_50_, and CC_70_) in 96-well plates in DMEM medium supplemented with 1% FBS and 1% antibiotics. Cells were stained with MitoSOX Red (1 μM) and 100 nΜ MitoTracker™ Green (Thermo Fisher Scientific, USA) at 37 °C for 30 min. Cells were washed twice with PBS, and fluorescence intensity was measured using a fluorescence microplate reader (Infinite M200 PRO, Tecan) at 396/610 nm (ex/em) and 490/516 nm (ex/em). The fluorescence intensity of MitoSOX Red was normalized using the Mitotracker Green intensity [65]. 

#### 4.1.7. RNA Extraction and cDNA Synthesis

Huh7 cells were treated for 48 h with the respective IsoB CC_30_ and CC_50_ concentrations. Control cells were also used. Total RNA was isolated from cells using Monarch^®^ Total RNA Miniprep Kit according to the manufacturer’s instructions. RNA integrity was determined by agarose gel electrophoresis, and concentration was measured spectrophotometrically by ND-1000 UV-VIS Spectrophotometer (Thermo Fisher Scientific, USA). For cDNA synthesis, 1 μg of total RNA was used for reverse transcription according to Super-Script II protocol instructions. 

#### 4.1.8. Real-Time Polymerase Chain Reaction (qPCR)

To study the transcript accumulation of human cancer-related genes, RT-qPCR was performed. The qPCR reactions were carried out using SYBR Select Master Mix, target gene primers (Table 1), and cDNA samples on StepOnePlus Real-Time PCR System (Applied Biosystems, Foster City, CA, USA). The PCR parameters were 95 °C for 10 minutes, 40 cycles of 95 °C for 15 s, and 60 °C for 1 min. The forward and reverse sequences of the target and housekeeping genes (GAPDH and ACTB) were designed using the PrimerExpress 2.0 software (Thermo Fisher Scientific, Waltham, MA, USA), and the relative transcript levels of the target genes were calculated by the delta-delta Ct(ΔΔCt) method [66]. Amplification efficiency (E) was determined using the LinReg PCR program [67]. Statistical differences were identified using one-way analysis of variance (ANOVA), followed by Tukey’s multiple comparisons test. All qPCRs were performed on 3 independent samples, and values were expressed as means ± SE.

#### 4.1.9. Data Analysis and Statistics

The graphical representations were crafted using SigmaPlot 12.0 software by Systat Software. Statistical analyses were performed utilizing the STATISTICA 12.0 software package (StatSoft Inc., Tulsa, OK, USA). To assess statistical significance, one-way ANOVA was employed at a confidence level of 95% (*p* < 0.05).

## 5. Conclusions

Based on the findings of this study, the compound IsoB emerges as a promising agent for liver cancer treatment, exhibiting both time- and dose-dependent cytotoxic effects. The effectiveness of IsoB is reinforced by its selective cytotoxic effect against cancerous liver cells. Furthermore, investigation into its cytotoxic mechanism revealed a significant negative impact on mitochondrial integrity and redox balance. Transcriptome analysis provided further insights into the molecular mechanism underlying IsoB cytotoxic activity in Huh7 cells, highlighting apoptosis-induced cytotoxicity combined with the regulation of genes related to liver cancer. However, it is important to note that further research is necessary to definitively establish IsoB’s suitability for cancer treatment. Further investigation into the specific molecular pathways and biomarker gene expression of liver cancer cells in response to 4-nitrophenyl-isothiazolones will shed light on their function and fortify their potential role in liver cancer treatment. Additionally, exploring the potential co-application of IsoB with existing anticancer agents like 5-FU could offer a strategy to overcome drug chemoresistance, paving the way for the development of novel and effective treatments for hepatocellular carcinoma.

## Figures and Tables

**Table 1:** Gene names and forward (F) and reverse (R) primer sequences.

Gene Symbol	Gene Name	Primer F (5′-3′)	Primer R (5′-3′)
ACTB	actin, beta	CTGTCCACCTTCCAGCAGATGT	AGCATTTGCGGTGGACGAT
BAX	BCL2-associated X protein	CGCCCTTTTCTACTTTGCCAG	GCCCATGATGGTTCTGATCAG
BID	BH3 interacting domain death agonist	TCCTTGCTCCGTGATGTCTTTC	AAGCTCCTCACGTAGGTGCGTA
BIRC3	baculoviral IAP repeat containing 3	TGCTTTTGCTGTGATGGTGG	CACTTGGCATGTTGAACCCA
CASP3	caspase 3	TGGAATTGATGCGTGATGTT	TGGCTCAGAAGCACACAAAC
CASP7	caspase 7	CTCTTCGCCTATTCCACGGTTC	TTGCACAAACCAGGAGCCTCT
CASP9	caspase 9	TGGACGACATCTTTGAGCAG	GCATTAGCGACCCTAAGCAG
CD44	CD44 molecule	TCATCTTGGCATCCCTCTTG	CCATTGCCACTGTTGATCACTA
FAS	Fas cell surface death receptor	GTGAACACTGTGACCCTTGCAC	TTGGTGTTGCTGGTGAGTGTG
GAPDH	glyceraldehyde-3-phosphate dehydrogenase	TTGCCCTCAACGACCACTTT	CACCCTGTTGCTGTAGCCAAA
MYCN	MYCN proto-oncogene, bHLH transcription factor	CCCCTGGGTCTGCCCCGTTT	GCCGAAGTAGAAGTCATCTT
TP53	tumor protein p53	CCAGCCAAAGAAGAAACCACTG	TCAGCTCTCGGAACATCTCGA

## Data Availability

Data are contained within the article.
